# Effects of age, gender, and hemisphere on cerebrovascular hemodynamics in children and young adults: Developmental scores and machine learning classifiers

**DOI:** 10.1371/journal.pone.0263106

**Published:** 2022-02-04

**Authors:** Marie Arsalidou, Nikolay Skuratov, Evgeny Khalezov, Alexander Bernstein, Evgeny Burnaev, Maxim Sharaev

**Affiliations:** 1 HSE University, Moscow, Russian Federation; 2 York University, Toronto, Canada; 3 Research Center in AI, Skolkovo Institute of Science and Technology, Moscow, Russian Federation; 4 AIRI, Moscow, Russian Federation; IRCCS Foundation Stella Maris: IRCCS Fondazione Stella Maris, ITALY

## Abstract

A constant blood supply to the brain is required for mental function. Research with Doppler ultrasonography has important clinical value and burgeoning potential with machine learning applications in studies predicting gestational age and vascular aging. Critically, studies on ultrasound metrics in school-age children are sparse and no machine learning study to date has used color duplex ultrasonography to predict age and classify age-group. The purpose of our study is two-fold: first to document cerebrovascular hemodynamics considering age, gender, and hemisphere in three arteries; and second to construct machine learning models that can predict and classify the age and age-group of a participant using ultrasonography metrics. We record peak systolic, end-diastolic, and time-averaged maximum velocities bilaterally in internal carotid, vertebral, and middle cerebral arteries from 821 participants. Results confirm that ultrasonography values decrease with age and reveal that gender and hemispheres show more similarities than differences, which depend on age, artery, and metric. Machine learning algorithms predict age and classifier models distinguish cerebrovascular hemodynamics between children and adults. Blood velocities, rather than blood vessel diameters, are more important for classifier models, and common and distinct variables contribute to age classification models for males and females.

## Introduction

Cerebrovascular function has a measurable impact on health and cognitive outcomes. Cerebrovascular hemodynamics rely on various measurements such as vessel diameter (i.e., arterial stenosis), and blood flow velocities (i.e., arterial pressure) [1]. Doppler ultrasonography provides non-invasive, rapid, and real-time values associated with cerebrovascular function and has established utility in clinical practice and research applications [2]. Medical conditions such as hypoxic-ischemic encephalopathy [3] and sickle cell disease [4], have been associated with altered cerebrovascular hemodynamics. Ultrasonography scores have been used with machine learning approaches to predict gestational age [5] and vascular aging [6]. Critically, developmental values related to blood vessels that supply the brain in school-age children are sparse and fragmented as information about gender, age, and hemisphere in major blood vessels is not consistently reported. As average developmental scores and predictions derived using machine learning algorithms can benefit clinical practice and future research, the purpose of this study is to examine the effects of age, gender, and hemisphere on ultrasound metrics from three major blood vessels (i.e., internal carotid, vertebral, and middle cerebral arteries) in a large sample of children and adults, and model using machine learning approach, find the features that better predict age and classify age-group.

The literature documents ranges of transcranial Doppler metrics in young adults [7] and across adulthood [8]. Two studies with larger samples of healthy adult participants [9,10] demonstrated that all individual vessel flows and total cerebral blood flow declined with age, at about 2.6 mL/min per year. Relatively fewer studies have investigated cerebrovascular hemodynamics in children. The first two studies with typically developing children that recorded blood flow velocities in basal cerebral arteries were published in 1988, the first included 25 participants under 20 years [11] and the second examined only 112 children showing that after 5–6 years of age, velocities decreased linearly [12]. These findings were later confirmed in different blood vessels by Schöning and Hartig [13] with a sample of 94 children. Critically, normative values in this study were reported in two age groups averaging values for children under 10 and children between 10 to 18 years. Subsequent studies verified and extended developmental data to include, for example, indices from 50 pre-school children ages 4, 5 and 6 years [14]. Some studies reported gender effects [9], whereas others did not [15]. A review that combines data from past studies [12,16,17], proposes normative bilateral values associated with age [18]. Although the review recognizes that gender is also a factor that influences blood flow velocities, developmental values by gender were not documented. Therefore, a need remains to better document and understand developmental effects as a function of hemisphere and gender.

Age dependency of cerebrovascular hemodynamics data may serve as a good factor for formulating machine learning models that can predict or classify age. Gender, hemisphere, and specific blood vessels may also relate differentially in age prediction and classification. Knowledge on the variables and biomarkers that are more critical for such predictions can be beneficial to targeted biometric research and decision support systems (e.g., computerized programs that facilitate decision making in institutions) such as for clinical, educational, and forensic sectors. Ultrasonography and machine learning have been used in medical research to predict gestational age [19,20] and biological age in aging adults [6]. The goal of our study was twofold: First, use Doppler ultrasonography to record cerebrovascular hemodynamics from internal carotid, vertebral, and middle cerebral arteries bilaterally, in a large sample of children and second use machine learning approaches to estimate the chronological age and classify children and adults highlighting also the most important contributing features of the model. Specifically, we hypothesized that age is negatively correlated with blood flow velocities. We did not anticipate any effects due to gender, and our investigation related to hemispheres was exploratory. Based on past machine learning algorithms using cerebral biometrics we hypothesized that models using head and neck ultrasonography metrics would be accurately predicting age and classifying age-groups. Feature importance related to robust models (i.e., which variable was contributed more to computational models) was exploratory.

## Materials and methods

### Participants

Participants were children and young adults (N = 821; 380 females, 441 males, age range 6–25 years). Children were recruited from eight urban public schools in Moscow that agreed to collaborate as part of a larger project during the school year between September 2017 and May 2019. The study followed a quasi-experimental design. Adults were recruited from the community. Table 1 shows age (calculated using date of birth) and gender distribution for nine age groups (i.e., 6–7 years, 8 years, 9 years, 10 years, 11 years, 12 years, 13–15 years, 16–19 years, and 20–25 years). Adult participants or children’s parents provided signed informed consent; children provided verbal assent. We did not screen for medical conditions, because the educational system in Russia offers education and social support for children with disabilities and neurodevelopmental disorders in specialized institutions [21]. Parents, teachers or school psychologists did not self-report any medical conditions. Other than attending classes in regular public schools we did not have specific inclusion or exclusion criteria. The research ethics board at HSE University approved all procedures.

**Table 1:** Developmental metrics of cerebrovascular hemodynamics by age group, gender, and hemisphere.

Left Hemisphere													
			MCA			ICA				VA			
6–7 years		age	TAMAX	eDV	pSV	TAMAX	eDV	pSV	d	TAMAX	eDV	pSV	d
T = 64	Mean	7.52	**101.80**	**69.67**	**141.56**	67.28	44.04	105.54	**4.32**	38.29	23.34	68.65	**3.50**
	SD	0.32	16.42	12.51	19.19	11.86	8.13	11.85	0.29	8.69	5.92	14.99	0.52
M = 37	Mean	7.55	**98.88**	67.74	**138.24**	67.25	44.21	104.81	4.35	38.13	**23.55**	69.81	3.58
	SD	0.30	13.84	10.88	16.54	12.88	8.62	12.68	0.24	6.17	4.59	10.93	0.55
F = 27	Mean	7.48	105.81	72.33	**146.11**	67.32	43.80	106.54	4.29	38.51	23.06	67.06	3.40
	SD	0.34	18.96	14.23	21.83	10.54	7.56	10.77	0.35	11.40	7.46	19.36	0.47
**8 years**													
T = 176	Mean	8.53	**99.97**	**69.17**	**137.52**	65.77	43.76	100.34	**4.24**	**38.13**	**23.59**	**64.65**	**3.43**
	SD	0.26	14.52	11.33	16.71	12.53	8.02	12.18	0.37	9.49	6.63	13.88	0.52
M = 78	Mean	8.55	**100.32**	**69.19**	**138.12**	68.15	45.48	101.89	**4.37**	**39.05**	**23.78**	**66.84**	**3.56**
	SD	0.27	15.03	11.51	16.92	14.17	8.88	12.20	0.34	9.69	7.27	14.64	0.51
F = 98	Mean	8.51	99.69	69.16	**137.04**	63.88	42.39	99.10	**4.13**	37.40	23.44	**62.90**	**3.33**
	SD	0.24	14.17	11.24	16.62	10.75	7.03	12.08	0.36	9.31	6.10	13.06	0.51
**9 years**													
T = 247	Mean	9.48	**97.95**	**68.03**	**134.85**	66.34	43.71	100.85	**4.34**	**37.62**	**23.56**	**64.06**	**3.42**
	SD	0.28	14.02	11.15	15.48	12.14	8.75	11.48	0.35	8.84	5.92	13.86	0.51
M = 135	Mean	9.46	**97.12**	**67.15**	**133.03**	67.85	44.66	101.82	4.44	**38.58**	**24.33**	**65.40**	**3.52**
	SD	0.28	14.07	11.36	14.33	12.16	8.45	11.85	0.34	9.19	5.95	14.03	0.48
F = 112	Mean	9.50	**98.95**	**69.09**	**137.04**	64.52	42.56	99.69	**4.22**	36.47	22.63	62.43	**3.30**
	SD	0.29	13.96	10.85	16.57	11.93	9.01	10.94	0.33	8.28	5.77	13.54	0.52
**10 years**													
T = 173	Mean	10.50	**96.89**	**68.68**	**133.92**	63.71	42.56	98.70	**4.35**	**37.32**	**23.46**	**64.68**	**3.38**
	SD	0.27	14.81	11.87	16.71	11.74	8.46	12.95	0.33	7.75	5.11	13.61	0.53
M = 102	Mean	10.53	**95.66**	**67.68**	**131.70**	66.48	44.40	100.46	4.42	**38.65**	**24.32**	**66.11**	3.46
	SD	0.27	15.44	12.31	17.30	12.17	8.80	12.97	0.34	8.17	5.15	14.20	0.58
F = 71	Mean	10.45	**98.65**	**70.11**	**137.11**	59.74	39.93	96.17	4.25	**35.41**	**22.22**	**62.63**	3.28
	SD	0.27	13.77	11.13	15.38	9.89	7.24	12.59	0.30	6.71	4.83	12.53	0.44
**11 years**													
T = 41	Mean	11.37	91.50	65.53	**131.05**	62.17	41.60	97.84	**4.34**	**37.42**	23.84	**64.28**	**3.43**
	SD	0.32	16.38	12.51	18.21	10.52	6.92	10.58	0.29	8.87	6.55	14.39	0.57
M = 25	Mean	11.36	89.97	64.65	129.00	62.35	42.08	99.59	4.36	**37.92**	24.14	**64.64**	3.45
	SD	0.28	17.23	12.44	18.12	10.09	6.76	9.85	0.26	9.27	6.96	15.46	0.51
F = 16	Mean	11.39	93.90	66.90	134.25	61.88	40.85	95.10	4.32	36.64	23.37	63.70	3.39
	SD	0.37	15.19	12.89	18.47	11.48	7.31	11.42	0.35	8.43	6.04	13.00	0.66
**12 years**													
T = 33	Mean	12.39	89.00	**63.52**	**128.88**	59.53	39.14	101.03	4.19	33.81	21.11	61.81	3.25
	SD	0.30	12.25	9.19	12.84	9.46	7.09	10.67	0.33	7.57	5.75	11.33	0.58
M = 19	Mean	12.45	87.59	61.00	127.16	59.62	39.60	103.51	4.27	33.46	20.13	60.44	3.34
	SD	0.30	14.46	9.81	13.29	9.01	7.15	9.48	0.32	7.58	5.42	9.40	0.55
F = 14	Mean	12.30	90.90	**66.93**	**131.21**	59.41	38.52	97.67	4.09	34.29	22.44	63.67	3.12
	SD	0.27	8.54	7.28	12.30	10.39	7.24	11.61	0.33	7.82	6.12	13.68	0.61
**13–15 years**													
T = 41	Mean	14.67	84.06	57.83	122.99	56.83	37.09	96.40	4.33	30.50	19.25	**57.26**	3.28
	SD	0.54	12.88	9.18	14.94	8.15	5.85	12.85	0.29	7.35	5.81	15.20	0.52
M = 22	Mean	14.60	81.58	55.60	120.96	57.20	36.24	101.83	4.41	31.61	19.25	63.53	3.40
	SD	0.57	13.29	9.72	15.00	7.50	3.99	10.14	0.26	8.78	6.80	17.36	0.50
F = 19	Mean	14.76	86.93	60.42	125.34	56.39	38.07	90.12	4.24	29.20	19.24	50.01	3.15
	SD	0.49	12.09	7.99	14.93	9.04	7.45	13.01	0.31	5.18	4.58	7.61	0.51
**16–19 years**													
T = 19	Mean	17.31	88.49	**63.53**	**127.32**	51.37	34.39	93.56	4.21	28.63	17.55	51.47	3.04
	SD	1.42	10.35	6.15	11.23	6.10	5.56	10.25	0.31	4.37	3.66	8.24	0.61
M = 8	Mean	17.30	84.40	61.33	125.38	49.44	33.11	92.21	4.28	28.66	17.78	51.21	3.15
	SD	1.55	12.21	7.24	9.94	6.11	4.72	11.63	0.29	4.44	4.72	8.36	0.70
F = 11	Mean	17.32	91.47	65.14	128.73	52.78	35.33	94.55	4.15	28.60	17.39	51.65	2.95
	SD	1.39	8.08	4.97	12.35	5.98	6.15	9.59	0.34	4.53	2.92	8.56	0.55
**20–25 years**													
T = 27	Mean	21.92	78.79	56.26	117.67	48.33	32.64	80.32	4.39	**31.30**	19.29	**55.00**	**3.41**
	SD	1.31	14.06	9.33	17.58	9.04	6.94	13.40	0.37	4.98	3.77	8.76	0.53
M = 15	Mean	22.73	72.39	52.40	109.67	46.29	31.37	75.69	4.51	**31.40**	19.59	**53.29**	3.47
	SD	1.16	13.48	9.05	19.05	8.66	6.60	10.52	0.36	5.16	4.65	7.10	0.61
F = 12	Mean	20.90	86.78	61.08	127.67	50.89	34.24	86.11	4.25	31.18	18.92	**57.14**	3.34
	SD	0.57	10.51	7.49	8.51	9.22	7.31	14.76	0.35	4.98	2.43	10.40	0.43
**Right hemisphere**													
			**MCA**			**ICA**				**VA**			
**6–7 years**		**age**	**TAMAX**	**eDV**	**pSV**	**TAMAX**	**eDV**	**pSV**	**d**	**TAMAX**	**eDV**	**pSV**	**d**
T = 64	Mean	7.52	*93*.*83*	*64*.*40*	*130*.*10*	69.06	46.00	104.99	*4*.*16*	34.95	20.78	63.30	*3*.*22*
	SD	0.32	16.53	12.21	18.80	13.42	9.50	13.64	0.31	9.76	6.40	16.16	0.53
M = 37	Mean	7.55	*89*.*93*	62.63	*126*.*54*	71.64	47.63	106.53	4.21	34.35	*19*.*98*	63.81	3.34
	SD	0.30	11.92	10.87	14.35	13.49	8.72	13.02	0.31	8.22	4.42	15.77	0.53
F = 27	Mean	7.48	99.16	66.82	*134*.*97*	65.52	43.75	102.88	4.09	35.79	21.88	62.60	3.06
	SD	0.34	20.35	13.68	22.99	12.73	10.22	14.43	0.28	11.67	8.38	16.96	0.49
**8 years**													
T = 176	Mean	8.53	*94*.*34*	*65*.*55*	*129*.*33*	64.22	43.90	98.25	*4*.*12*	*34*.*46*	*21*.*09*	*58*.*57*	*3*.*14*
	SD	0.26	14.76	11.37	16.11	11.78	8.47	12.15	0.36	7.72	5.15	12.95	0.49
M = 78	Mean	8.55	*92*.*12*	*64*.*59*	*127*.*47*	65.62	44.83	99.99	*4*.*23*	*34*.*68*	*20*.*78*	*59*.*52*	*3*.*33*
	SD	0.27	14.49	11.50	15.71	11.71	8.24	11.20	0.33	8.20	5.05	12.63	0.46
F = 98	Mean	8.51	96.11	66.32	*130*.*81*	63.11	43.16	96.86	*4*.*02*	34.29	21.34	*57*.*82*	*2*.*99*
	SD	0.24	14.80	11.26	16.36	11.78	8.63	12.74	0.36	7.35	5.23	13.23	0.47
**9 years**													
T = 247	Mean	9.48	*90*.*45*	*62*.*63*	*125*.*69*	64.71	43.63	99.41	*4*.*25*	*34*.*28*	*21*.*34*	*58*.*94*	*3*.*21*
	SD	0.28	12.90	10.27	14.51	10.83	7.59	11.02	0.36	9.01	5.67	13.91	0.48
M = 135	Mean	9.46	*89*.*94*	*62*.*68*	*124*.*24*	66.05	44.59	100.82	4.35	*34*.*06*	*21*.*35*	*59*.*43*	*3*.*29*
	SD	0.28	13.01	10.37	13.98	10.43	7.39	10.67	0.33	9.20	5.62	13.68	0.47
F = 112	Mean	9.50	*91*.*06*	*62*.*56*	*127*.*45*	63.09	42.47	97.71	*4*.*12*	34.54	21.32	58.36	*3*.*11*
	SD	0.29	12.80	10.20	15.00	11.13	7.71	11.25	0.35	8.80	5.75	14.21	0.47
**10 years**													
T = 173	Mean	10.50	*90*.*00*	*62*.*85*	*125*.*33*	63.08	43.22	97.25	*4*.*25*	*32*.*31*	*19*.*95*	*55*.*51*	*3*.*20*
	SD	0.27	14.07	10.94	16.35	11.23	8.43	12.20	0.35	7.81	4.93	12.24	0.53
M = 102	Mean	10.53	*88*.*98*	*61*.*79*	*123*.*83*	64.47	44.42	97.58	4.33	*33*.*07*	*20*.*43*	*56*.*40*	3.31
	SD	0.27	13.85	11.13	16.45	11.96	8.97	12.48	0.33	8.34	5.11	12.55	0.56
F = 71	Mean	10.45	*91*.*47*	*64*.*38*	*127*.*49*	61.07	41.49	96.77	4.14	*31*.*22*	*19*.*26*	*54*.*24*	3.05
	SD	0.27	14.34	10.56	16.08	9.84	7.31	11.87	0.36	6.89	4.61	11.75	0.43
**11 years**													
T = 41	Mean	11.37	85.82	60.36	*121*.*50*	62.89	41.57	96.98	*4*.*17*	*32*.*37*	20.52	*55*.*92*	*3*.*07*
	SD	0.32	13.18	9.33	10.93	10.66	7.98	11.85	0.40	6.46	4.28	9.92	0.40
M = 25	Mean	11.36	83.98	59.01	120.34	63.78	42.41	98.57	4.30	*32*.*09*	20.27	*55*.*56*	3.12
	SD	0.28	12.14	9.32	11.38	11.24	8.59	12.48	0.29	5.81	3.95	9.43	0.42
F = 16	Mean	11.39	88.71	62.46	123.31	61.51	40.25	94.50	3.96	32.82	20.91	56.49	2.99
	SD	0.37	14.60	9.23	10.27	9.88	6.97	10.69	0.47	7.56	4.86	10.94	0.37
**12 years**													
T = 33	Mean	12.39	82.81	*57*.*99*	*122*.*38*	59.31	39.38	98.60	4.15	29.75	19.08	56.08	3.25
	SD	0.30	13.87	10.48	13.59	9.28	7.51	12.58	0.30	6.92	4.23	14.39	0.50
M = 19	Mean	12.45	82.59	56.97	121.98	60.01	39.42	99.61	4.15	29.10	18.64	55.33	3.22
	SD	0.30	15.73	12.39	15.52	8.71	6.96	12.73	0.35	6.95	3.74	14.67	0.61
F = 14	Mean	12.30	83.10	*59*.*37*	*122*.*93*	58.36	39.34	97.24	4.16	30.64	19.69	57.09	3.28
	SD	0.27	11.42	7.35	10.97	10.25	8.49	12.72	0.24	7.05	4.90	14.48	0.33
**13–15 years**													
T = 41	Mean	14.67	80.62	56.26	118.56	52.69	34.39	91.62	4.14	26.17	16.30	*48*.*57*	3.02
	SD	0.54	12.39	11.18	14.98	8.93	6.54	15.47	0.44	8.07	5.54	15.50	0.62
M = 22	Mean	14.60	76.90	53.33	115.26	53.18	34.82	96.64	4.31	27.68	16.82	54.35	3.10
	SD	0.57	12.70	11.22	15.32	7.52	5.07	14.65	0.46	7.62	5.81	14.66	0.66
F = 19	Mean	14.76	84.93	59.65	122.37	52.11	33.88	85.82	3.93	24.43	15.69	41.88	2.93
	SD	0.49	10.78	10.41	14.00	10.51	8.04	14.68	0.32	8.42	5.30	13.96	0.58
**16–19 years**													
T = 19	Mean	17.31	79.98	*56*.*17*	*117*.*32*	51.16	34.09	91.77	4.23	30.30	19.86	52.40	3.36
	SD	1.42	14.78	10.75	16.33	9.59	7.11	15.53	0.47	7.62	6.10	13.85	0.43
M = 8	Mean	17.30	74.04	53.43	111.80	52.48	33.45	95.25	4.44	28.65	19.06	49.88	3.35
	SD	1.55	13.21	7.41	15.32	8.76	5.79	15.54	0.39	6.65	4.49	14.41	0.40
F = 11	Mean	17.32	84.30	58.17	121.34	50.21	34.55	89.24	4.08	31.50	20.45	54.24	3.37
	SD	1.39	14.91	12.61	16.54	10.46	8.18	15.77	0.48	8.35	7.21	13.82	0.46
**20–25 years**													
T = 27	Mean	21.92	75.79	53.20	113.50	47.07	31.89	78.69	4.24	*24*.*51*	16.01	*43*.*99*	*2*.*88*
	SD	1.31	14.85	11.01	17.76	10.02	6.27	13.13	0.42	4.86	3.21	7.81	0.53
M = 15	Mean	22.73	68.16	47.89	103.16	45.27	30.79	77.61	4.41	*23*.*98*	15.82	*42*.*59*	2.95
	SD	1.16	13.38	9.30	14.95	7.00	4.56	12.47	0.30	4.76	3.72	7.69	0.58
F = 12	Mean	20.90	85.32	59.83	126.42	49.32	33.26	80.03	4.02	25.18	16.25	*45*.*73*	2.80
	SD	0.57	10.73	9.50	11.55	12.85	7.93	14.35	0.45	5.11	2.58	7.93	0.47

MCA = middle cerebral artery; ICA = internal carotid artery; VA = vertebral artery; TAMAX = time-averaged maximum flow velocity; eDV = end diastolic velocity; pSV = peak systolic velocity; d = vessel diameter; T = total number of participants; M = number of male participants; F = number of female participants; SD = standard deviation; Bold or Italics font = statistically significant difference between hemispheres at p = 0.05 corrected for multiple comparisons using Bonferroni, bold indicates the hemisphere with the largest value; orange = females have statistically significant higher scores than males at p = 0.05 corrected for multiple comparisons using Bonferroni; blue = males have statistically significant higher scores than females at p = 0.05 corrected for multiple comparisons using Bonferroni. All velocities were measured in cm/s and all vessel diameters were measured in mm.

### Measurements

**Fig 1:**
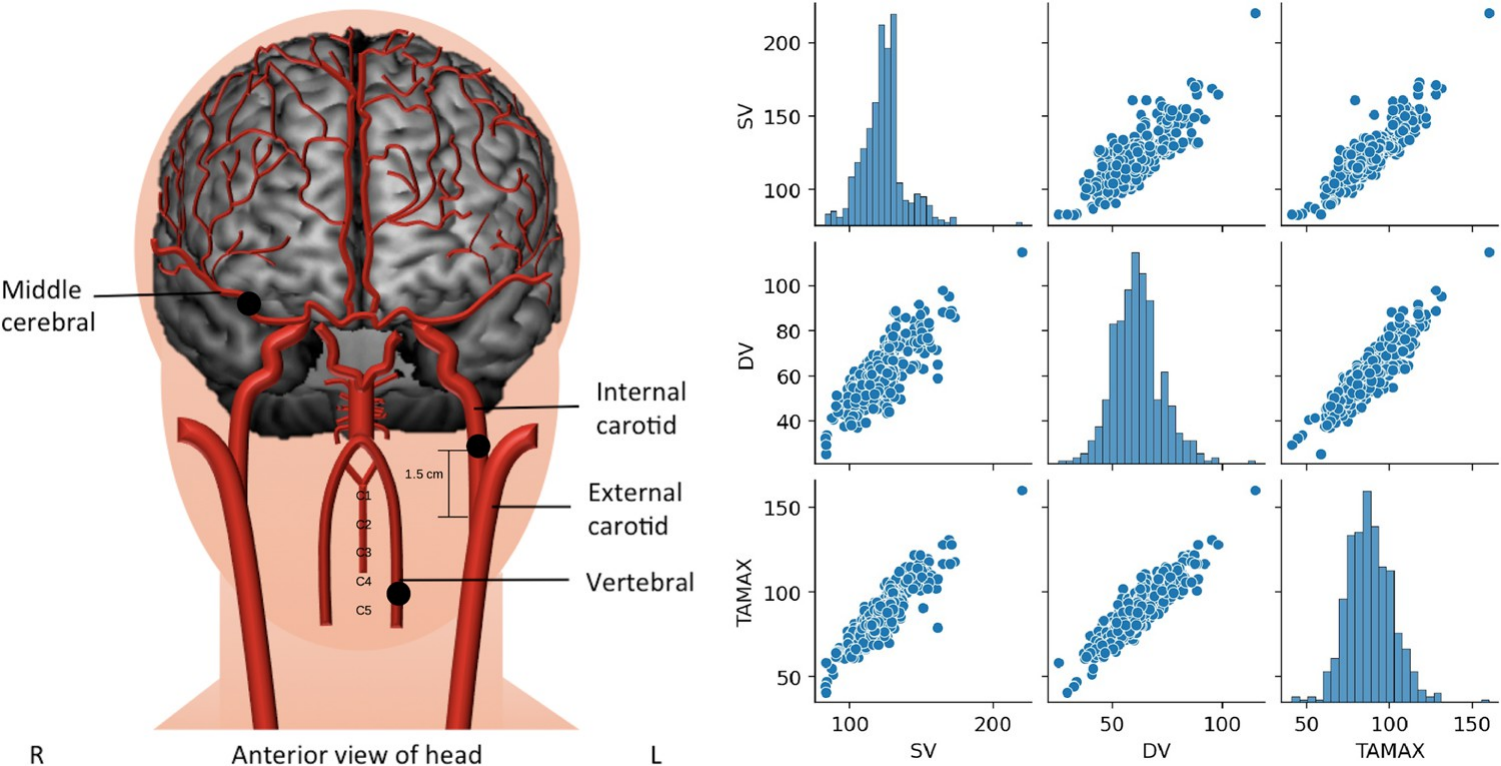
Anatomy of cerebral vasculature with approximate locations of measurement and example of doppler ultrasonography dependencies in the right middle cerebral artery. On the left, for the middle cerebral arteries measurements are taken in the M1 segment. For the internal carotid arteries measurements are taken 1.5 cm distal to the bifurcation (i.e., where the common carotid artery divides into the internal and external carotid arteries) for the internal carotid artery. For the vertebral arteries measurements are taken in the C4-C5 inter-transverse segment (between the C4 and C5 vertebrae). On the right, we illustrate doppler dependencies among peak systolic velocities (SV), end-diastolic velocities (DV), and time-averaged maximum (TAMAX) velocities. Histograms are on the diagonal and scatter plots for corresponding pairs of features are given off the diagonal.

Parameters of cerebrovascular hemodynamics were measured by means of transcranial color duplex ultrasonography. Measurements were recorded using a SonoSite M-Turbo ultrasound machine (FUJIFILM SonoSite, Inc., Bothell, WA, USA) for both extracranial and transcranial recordings. We used a L38x 10–5 MHz transducer probe to determine blood vessel diameters and blood velocities of the internal carotid arteries and vertebral arteries and P21x 5–1 MHz transducer probe on the transtemporal window to determine blood velocities of the middle cerebral arteries in M1 segment. Fig 1 illustrates approximate locations of recordings and some examples of dependencies between measured features of the right middle cerebral artery. Internal carotid arteries and vertebral arteries were evaluated bilaterally with high-resolution B-mode ultrasonography. Peak systolic (pSV), end-diastolic (eDV), and time-averaged maximum (TAMAX) velocities were measured using extracranial Doppler for internal carotid arteries (ICA) and vertebral arteries (VA), and using transcranial color duplex sonography for middle cerebral arteries (MCA). All velocities were measured in cm/s and all vessel diameters were measured in mm. Measurements were recorded in real-time, no images were collected.

### Procedure

Children were individually examined by means of duplex ultrasonography at their school. The sonography protocol was approximately 10 minutes and was completed in one session We used imaging ultrasonography while participants were laying down. The study was performed via scanning in color and pulse-wave Doppler mode. Diameters of the internal carotid arteries and vertebral arteries were measured based on the image in B-mode and color Doppler. Pulsed Doppler was used for the assessment of blood velocity in internal carotid arteries, vertebral arteries, and middle cerebral arteries. Several consistently repeated, almost identical waveforms were visualized and then their values were measured. The quantitative assessment of spectrum of Doppler shift was conducted by the peak systolic, maximum end-diastolic, and time-averaged maximum blood flow velocity. All measurements were recorded by a single experienced sonographer using a single ultrasound scanner and standardized protocol; thus, no inter-rater variability was estimated. We did not screen for medical conditions and no other vital signs (e.g., temperature, blood pressure) were recorded.

#### Statistical analyses

Descriptive and inferential statistics were calculated using SPSS (IBM SPSS Statistics 26). Descriptive statistics (mean and standard deviation) were derived as a function of age, gender, and hemisphere. Bivariate correlations (Pearson’s r, and Spearman’s ρ (rho)), within group, and between group sample contrasts (corrected using Bonferroni, α = 0.05) were performed to examine age, hemispheric, and gender effects, respectively. Normality was examined for all ultrasound metrics by age group using the Kolmogorov-Smirnov Test (corrected using Bonferroni, α = 0.05; n = 22 variables for each age group; 163 of 198 tests (i.e., 82%) were normally distributed). Pearson’s correlations that examine linear relations do not assume normality. Similarly, standard machine learning algorithms for construction of predictive models do not rely on the normality assumption of data [22].

Before applying machine learning algorithms, we preprocessed the data: removed the mean value, scaled each feature to unit variance separately and checked to identify features with non-zero variance in order to avoid having features with the same or almost the same values remaining in the dataset. The whole procedure is common and useful for many machine learning algorithms such as Logistic Regression [23], K-Nearest Neighbours Classifier [24], Support Vector Classifier [25]. Also, the dataset was screened for missing values. Missing data values (3.6%) mainly related to velocities in middle cerebral arteries were due to temporal bone thickness (i.e., although the temporal bone window is the thinnest area of the lateral skull, some participants had insufficient acoustic temporal bone window to insonate the circulation in the middle cerebral artery). We chose to account for missing values because some age groups have a modest number of participants. To account for missing values in the dataset, we tested several imputation methods to generate scores that replace those values; the most common methods include replacement with mean, median, mode, and K-Nearest Neighbours (KNN)—that assigns a value to the missing cell based on the nearest neighbours [26]. Although all imputation methods yielded comparable results, the best method to replace missing values was the mode of the feature, likely because of the small number of missing values. We confirmed that data processing pipelines where mode imputation method was selected showed better results in classification and regression tasks, with F1 score for classification and Mean Absolute Error for regression quality metric.

Exploratory data analysis was performed to find hidden patterns in the dataset using special visualization and statistical tools [27]. In particular, a correlation matrix among features was computed [28] to detect highly correlated features (see Fig 1 for an example). Presence of such features in the dataset can lead to instability of machine learning models and can decrease the accuracy of predictions. The dataset was also split into males and females, thus, a total of three datasets were used for building models.

#### Machine learning approaches

Machine learning approaches were used to predict a child’s age and distinguish between children and adults, from hemodynamic responses recorded using ultrasonography. Data from each participant was represented by a feature vector that includes bilateral indices (e.g., peak systolic velocity, end-diastolic velocity, vessel diameter, time-averaged maximum velocity), from three main arteries that supply the cortex with blood. A total of 22 features related to ultrasonography metrics. Additional features indicating participant characteristics include gender, school grade and age. Some features, such as the school grade of the child, were redundant and posed a data leak in building the predictive model, because the age of a child can be accurately reconstructed from the school grade; thus, we removed/reduced such features.

First, we provide a regression analysis for age prediction. We used a standard pipeline of data processing [29]. The pipeline consisted of the following steps: feature selection and dimensionality reduction, various machine learning models fitting and their hyperparameters search. Models were evaluated in the process of cross-validation. More details on steps taken for the regression analysis are reported in supplementary materials (S1 Appendix).

#### Distinguishing children and adults with a binary classification task

Our sample consists of a large sample of younger children, and a modest sample of adolescents and adults. It was practically reasonable to combine adolescents and adults. This decision was also based on past research in biology and psychology. Specifically, research demonstrates that most biological maturation indices peak by the age of 16 years [30]. Theoretically, according to a developmental cognitive framework we expected that children of 15–16 year-olds would reach cognitive abilities (e.g., mental-attentional capacity) similar to young adults [31–33], giving justification to our choice of merging data from older adolescents with young adults. Therefore, to classify children and adults we adjusted our standard pipeline [29] of data processing for imbalanced classification tasks [34] due to unequal sample sizes in age groups.

First, we labeled participants according to their age in the following way: we introduced two classes—children (< 16 years old) and adults (≥ 16 years old). Next, we applied sampling methods to balance classes and a dimensionality reduction step. The dimensionality reduction step includes both feature extraction and feature selection procedures. To extract features we applied Principal Component Analysis (PCA) [35] and Locally Linear Embedding (LLE) [36]. For feature selection we used ready-to-use Python routines SelectKBest and SelectPercentile from the Scikit-learn library [37]. Following, we tested different machine learning models: Logistic Regression [23], K-Nearest Neighbours Classifier [24], XGBoost Classifier [38], Support Vector Classifier [25], Random Forest Classifier [39], and Gaussian Naïve Bayes Classifier [40]. We used a stratified cross-validation technique and used another accuracy score to evaluate classification results, namely, we used macro averaged F1 score, which can be defined as a weighted average of the precision and recall. Further, we applied a cross-validation approach called “Stratified K-fold” [41]. Its main concept is the same as of “Shuffled K-fold”, but in this approach the dataset was split preserving the initial percentage of each class. During the cross-validation procedure we applied an oversampling or undersampling technique to k-1 groups and fitted the proposed model on sampled data. Preferred approaches of oversampling and undersampling, which tend to show good results in practice were applied: Synthetic Minority Over-sampling Technique [42], Adaptive Synthetic [43], Random Over Sampler [44], Random Under Sampler [45], All K-Nearest Neighbors [46]. The impurity-based feature importances were calculated within Random Forest Classifier Python routine. Here the importance of a particular feature is computed as the normalized total reduction of the criterion brought by that feature [47].

## Results

**Fig 2:**
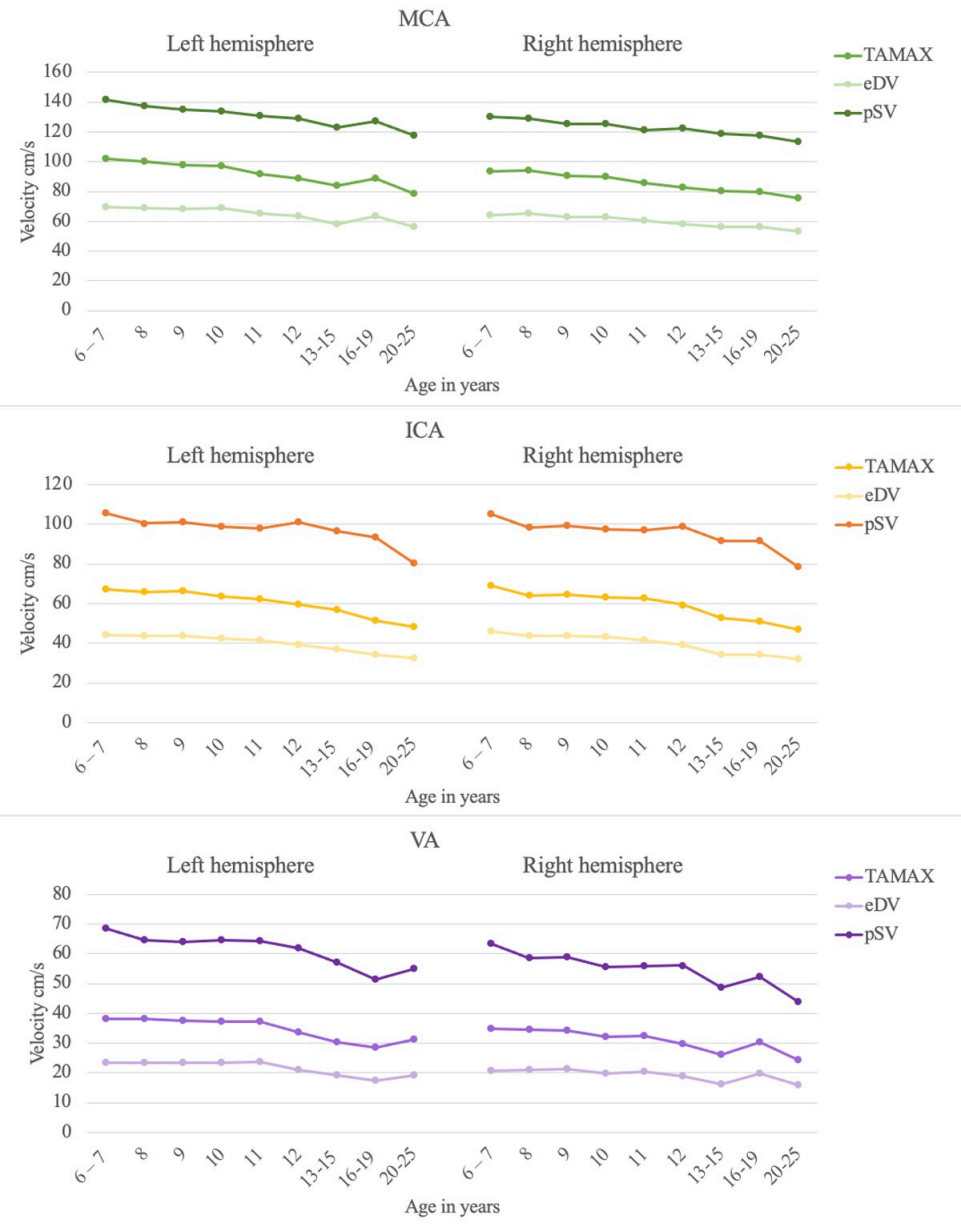
Velocities as a function of age, gender, blood vessel and hemisphere for the total sample. MCA = middle cerebral artery; ICA = internal carotid artery; VA = vertebral artery; TAMAX = time-averaged maximum flow velocity; eDV = end-diastolic velocity; pSV = peak systolic velocity.

**Fig 3:**
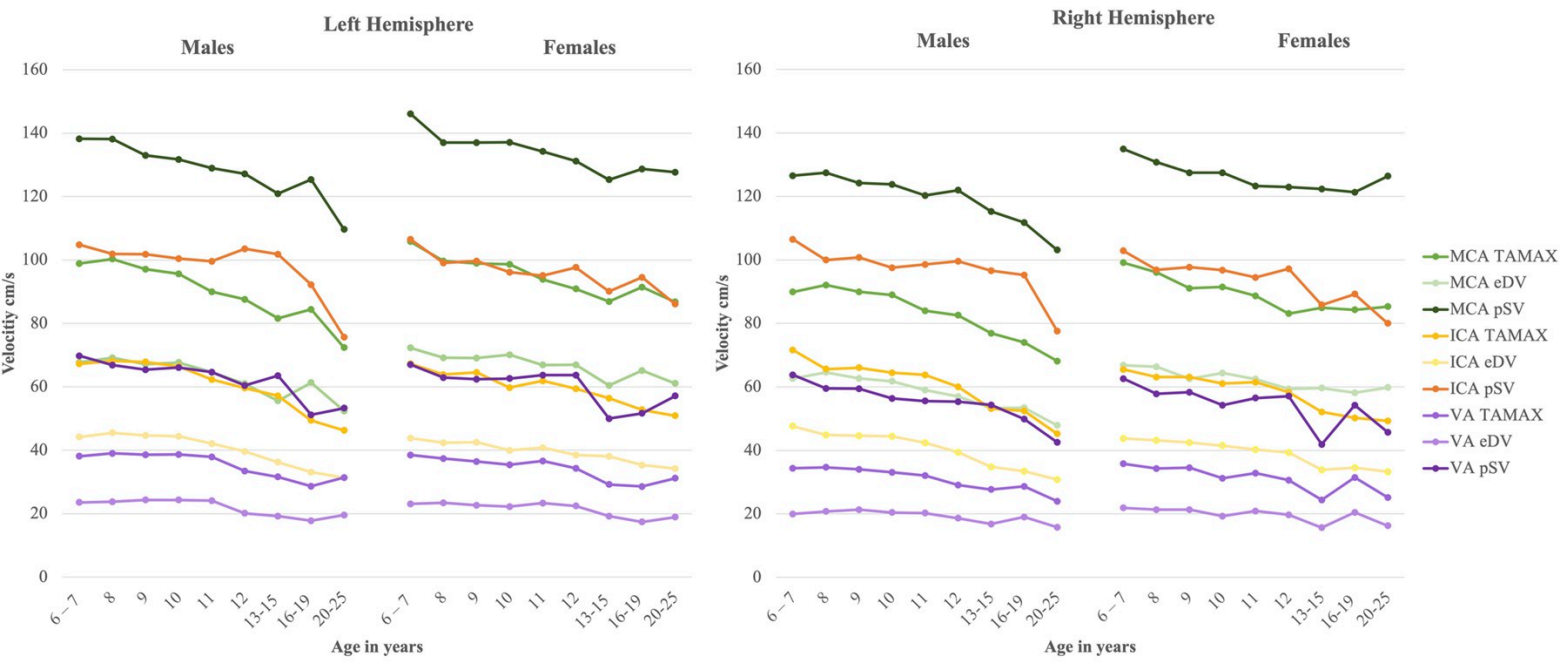
Velocities as a function of age, gender, blood vessel and hemisphere for males and females. MCA = middle cerebral artery; ICA = internal carotid artery; VA = vertebral artery; TAMAX = time-averaged maximum flow velocity; eDV = end-diastolic velocity; pSV = peak systolic velocity.

Data associated with blood vessel, age group, gender, and hemisphere are illustrated in Figs 2 and 3, and tabulated in Table 1. Between-group comparisons showing significant gender effects are marked with red when females show higher values and blue when males show higher values (Table 1). Within-group comparisons showing significant effects of the hemisphere are marked in green; all significant hemispheric effects show higher values in the left hemisphere (Table 1). Note that some metrics show both gender and hemispheric effects. Overall, there are more similarities between genders (~80% of comparisons show no significant differences), whereas about 40% of comparisons between hemispheres show significant differences; males show more hemispheric differences than females. Statistically significant correlations with age are observed for all variables, except for blood vessel diameters (Table 2). Negative relations indicate a decrease in velocities as a function of age, with shared variance ranging from 4.49% in the vertebral arteries to 13.6% in the internal carotid arteries.

**Table 2:** Correlations with age.

**Left Hemisphere**												
		**MCA**			**ICA**				**VA**			
		**TAMAX**	**DV**	**SV**	**TAMAX**	**DV**	**SV**	**d**	**TAMAX**	**DV**	**SV**	**d**
Total	r	-.342**	-.268**	-.290**	-.346**	-.316**	-.310**	0.025	-.242**	-.213**	-.212**	-0.089
	p-value	0.000	0.000	0.000	0.000	0.000	0.000	0.476	0.000	0.000	0.000	0.011
	R^2^	0.117	0.072	0.084	0.120	0.100	0.096		0.059	0.045	0.045	
	ρ (rho)	-.293**	-.210**	-.242**	-.273**	-.254**	-.207**	0.028	-.199**	-.167**	-.166**	-.093
	p-value	0.000	0.000	0.000	0.000	0.000	0.000	0.428	0.000	0.000	0.000	0.008
Males	r	-.391**	-.312**	-.347**	-.384**	-.368**	-.349**	0.021	-.247**	-.220**	-.235**	-0.095
	p	0.000	0.000	0.000	0.000	0.000	0.000	0.667	0.000	0.000	0.000	0.045
	R^2^	0.153	0.097	0.120	0.147	0.135	0.122		0.061	0.048	0.055	
	ρ (rho)	-.325**	-.245**	-.288**	-.283**	-.278**	-.195**	-0.001	-.191**	-.159**	-.183**	-.115*
	p-value	0.000	0.000	0.000	0.000	0.000	0.000	0.979	0.000	0.001	0.000	0.016
Females	r	-.273**	-.208**	-.215**	-.308**	-.260**	-.269**	0.015	-.246**	-.210**	-.193**	-0.097
	p	0.000	0.000	0.000	0.000	0.000	0.000	0.771	0.000	0.000	0.000	0.059
	R^2^	0.075	0.043	0.046	0.095	0.068	0.072		0.061	0.044	0.037	
	ρ (rho)	-.257**	-.168**	-.185**	-.289**	-.252**	-.239**	0.030	-.218**	-.183**	-.153**	-0.089
	p-value	0.000	0.001	0.000	0.000	0.000	0.000	0.565	0.000	0.000	0.003	0.083
**Right Hemisphere**												
		**MCA**			**ICA**				**VA**			
		**TAMAX**	**DV**	**SV**	**TAMAX**	**DV**	**SV**	**d**	**TAMAX**	**DV**	**SV**	**d**
Total	r	-.301**	-.250**	-.231**	-.369**	-.363**	-.323**	0.031	-.278**	-.213**	-.261**	-0.076
	p	0.000	0.000	0.000	0.000	0.000	0.000	0.380	0.000	0.000	0.000	0.029
	R^2^	0.091	0.063	0.053	0.136	0.132	0.104		0.077	0.045	0.068	
	ρ (rho)	-.258**	-.212**	-.191**	-.273**	-.275**	-.209**	.069	-.247**	-.164**	-.226**	-0.035
	p-value	0.000	0.000	0.000	0.000	0.000	0.000	0.050	0.000	0.000	0.000	0.323
Males	r	-.358**	-.309**	-.304**	-.419**	-.414**	-.351**	0.077	-.276**	-.209**	-.273**	-0.134
	p	0.000	0.000	0.000	0.000	0.000	0.000	0.105	0.000	0.000	0.000	0.005
	R^2^	0.128	0.095	0.092	0.176	0.171	0.123		0.076	0.044	0.075	
	ρ (rho)	-.268**	-.240**	-.212**	-.324**	-.316**	-.223**	0.087	-.224**	-.118	-.224**	-.106
	p-value	0.000	0.000	0.000	0.000	0.000	0.000	0.068	0.000	0.013	0.000	0.026
Females	r	-.230**	-.172**	-0.142	-.318**	-.312**	-.303**	-0.039	-.282**	-.218**	-.251**	-0.017
	p	0.000	0.001	0.006	0.000	0.000	0.000	0.453	0.000	0.000	0.000	0.736
	R^2^	0.053	0.030		0.101	0.097	0.092		0.080	0.048	0.063	
	ρ (rho)	-.246**	-.180**	-.159**	-.231**	-.242**	-.206**	0.012	-.276**	-.217**	-.236**	0.023
	p-value	0.000	0.000	0.002	0.000	0.000	0.000	0.822	0.000	0.000	0.000	0.652

** Significant at p < 0.0022 (p = 0.05 corrected for multiple comparisons using Bonferroni). MCA = middle cerebral artery; ICA = internal carotid artery; VA = vertebral artery; TAMAX = time-averaged maximum flow velocity; eDV = end-diastolic velocity; pSV = peak systolic velocity; d = vessel diameter.

### Predictive models

#### Regression results

Mean absolute error scores for machine learning models built to predict a child’s age are tabulated for all participants, and males and females separately (Table 1). The best predictive model with all participants could predict a child’s age within mean absolute error of 0.82 ± 0.06 (i.e. with accuracy about 10 months). The top performing machine learning model is based on Lasso Regression. Separate predictive models for males and females also had high predictive power with mean absolute error of 0.819 ±0.079 for males and 0.799 ± 0.099 for females. More details on the regression analyses can be found in Supplementary materials S1 Appendix.

#### Classification results

Top results associated with distinguishing age groups using machine learning classification models are listed in Table 3. We present results by machine learning task, considering experiments with and without oversampling and as a function of gender. The best model is obtained using the Gaussian Naive Bayes Classifier with mean F1 score of 0.67 ± 0.08. The best of the four classification models was obtained with sampling techniques. The best model in this experiment is obtained using Random Forest Classifier with SMOTE oversampling technique and the F1 score of 0.73 ± 0.04. By applying Random Forest Classifier with Random Oversampling Technique we achieve the F1 score of 0.77 ± 0.06 in males. The best model for females is the Random Forest Classifier without using sampling techniques; its F1 score is equal to 0.69 ± 0.17.

**Table 3:** Best classification models.

Model	Task	Mean F1	STD F1
GNB	Without sampling	**0.67**	**0.08**
KNC	Without sampling	0.66	0.08
RFC	Without sampling	0.62	0.09
LR	Without sampling	0.55	0.10
RFC + SMOTE	With sampling	**0.73**	**0.04**
RFC + ADS	With sampling	0.73	0.02
RFC + ROS	With sampling	0.65	0.03
GNB + SMOTE	With sampling	0.64	0.04
RFC + ROS	Male	**0.77**	**0.06**
KNC	Male	0.76	0.02
GNB	Male	0.75	0.11
RFC + ADS	Male	0.73	0.3
RFC	Female	**0.69**	**0.17**
RFC + ROS	Female	0.66	0.03
RFC + SMOTE	Female	0.64	0.11
RFC + ADS	Female	0.64	0.09

Classification methods: GNB—Gaussian Naïve Bayes Classifier, XGBC—XGBoost Classifier, RFC—Random Forest Classifier, KNC—K-Nearest Neighbours Classifier. Sampling methods: SMOTE—Synthetic Minority Over-sampling Technique, ROS—Random Oversampling, ADS—Adaptive Synthetic Over-sampling Technique. Tasks: Without Sampling—Experiments without sampling techniques, With sampling—Experiments with sampling techniques, Male—Experiments with male sample, Female—Experiments with Female sample. Mean F1, STD F1—mean value and standard deviation of F1 score values after all iterations of cross-validation are performed.

**Fig 4:**
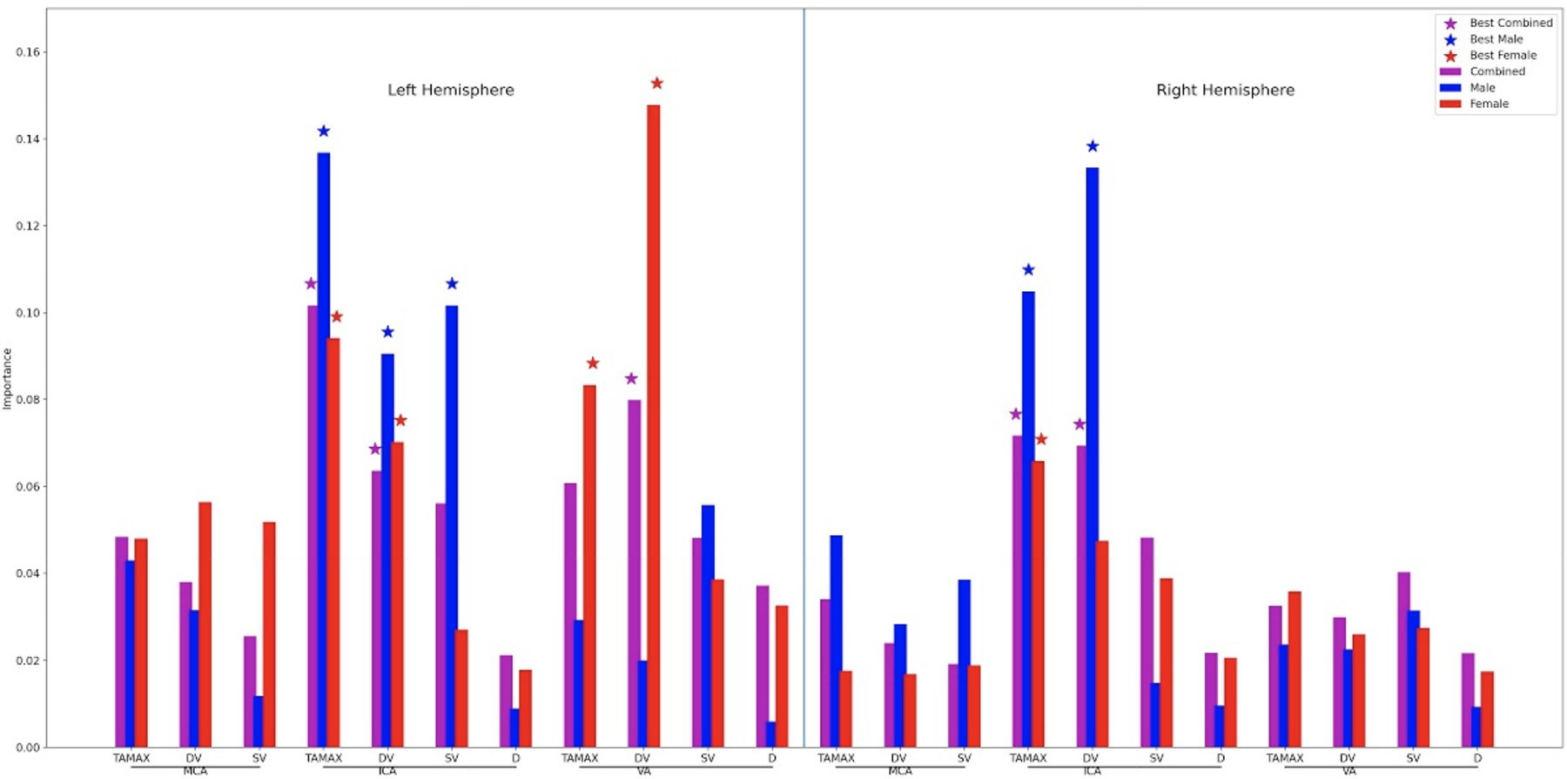
Feature importance of best models for the combined total sample, males and females. MCA = middle cerebral artery; ICA = internal carotid artery; VA = vertebral artery; TAMAX = time-averaged maximum flow velocity; eDV = end-diastolic velocity; pSV = peak systolic velocity; d = vessel diameter; * top five features.

To evaluate our machine learning models we provide Paired Stratified KFold t-test [48] based on 100 iterations for every combination of best models from each experiment at p = 0.05. Results show that the model for males is significantly different from the model for females (p = 0.001). The models for males and females are also significantly different from the model derived from all participants with p = 0.009 and p = 0.021, respectively. Feature importance values by group and hemisphere are illustrated on Fig 4.

## Discussion

Metrics associated with blood vessels that supply the brain are examined using transcranial and extracranial color duplex sonography. We report for the first time ultrasonography metrics of cerebrovascular hemodynamics from a large sample of school-aged children and young adults considering age, gender, and hemisphere in middle cerebral, internal carotid and vertebral arteries. Machine learning approaches are also used for the first time to predict and classify age and age groups in our sample. We highlight three main findings: (a) Results confirm age dependency of blood flow velocities, however hemispheric and gender effects depend on age group and blood vessel. Specifically, most hemispheric asymmetries are observed in children under 11 years old in the middle cerebral and vertebral arteries, and all significant hemispheric effects show higher values in the left hemisphere. Males and females show more similarities than differences in cerebrovascular hemodynamics; when significant differences were observed females showed higher values in all comparisons involving the middle cerebral artery, whereas males showed higher values in comparisons involving internal carotid and vertebral arteries with one exception in peak systolic velocity of the left internal carotid artery in young adults. (b) Although machine learning approaches for quantitative age prediction perform with low mean absolute error scores, this is likely due to a narrow continuous age range of children, as verified by simple models performing constant prediction (i.e. mean group age; please see supplementary material S1 Appendix). Machine learning classification models with and without sampling techniques distinguish children from young adults with high accuracy. Notably top models that distinguish age groups in males, females and with the total sample are different. (c) Results of feature importance show common and distinct features (i.e., ultrasonography metrics) that contribute highly to the classification models of cerebrovascular hemodynamics for males and females. Specifically, values in the internal carotid artery, particularly in the left hemisphere make high contributions to models of both males and females, whereas values in the vertebral arteries contributed highly to the model for females. This is consistent with elementary analyses showing higher age dependencies in internal carotid metrics and more gender and hemispheric differences in middle cerebral and vertebral arteries. Developmental values of cerebrovascular blood flow parameters and machine learning approaches that consider features of age, gender, and hemisphere may benefit clinical evaluations and future research.

Age significantly relates to blood flow velocities. The strongest relations with age are observed in the right internal carotid artery showing 13% of common variance in the total sample, about 17% of common variance for males and about 10% of common variance for females. The current results are consistent and replicate with a larger sample size results of previous empirical studies that document negative relations with age [10,13,49,50].

Hemispheric asymmetries are observed in about 40% of left versus right hemisphere comparisons of the total sample; males showed hemispheric asymmetries in 28% of comparisons, and females showed hemispheric asymmetries in 19% of comparisons. Blood flow velocities associated with the internal carotid artery reveal no significant hemispheric differences, whereas a few differences are observed in vessel diameters particularly in younger age groups. The majority of hemispheric differences are observed mainly in metrics associated with the middle cerebral and vertebral arteries with the left hemisphere showing higher values. Hemispheric asymmetries in these blood vessels are more prevalent in younger age groups, particularly in children under 11 years. These results add to past adult studies that examined hemispheric effects [10,49]. Some adult studies found no significant differences between hemispheres [51,52]. Krakauskaite [7] examined two age groups (14–19 and 20–29 year-olds) and found no differences between left- and right-side segments of the circle of Willis, with the exception of the distal M1 (p = 0.022) of the middle cerebral artery and the C1 (p < 0.0001) of the internal carotid artery, both showing higher velocities in the left hemisphere. Albayarak [49] in another study with adults showed hemispheric differences in blood flow volume in both vertebral and internal carotid arteries. Hemispheric asymmetries primarily favoring the left hemisphere with higher velocities certainly mark a topic for further research. We speculate that in school-aged children this asymmetry may be related to hemispheric synchronization showing alternating patterns between the left and right hemispheres as documented using electroencephalography [53,54]. Such patterns have been interpreted to correspond to developmental stages. Alternatively, also from a developmental perspective, hemispheric differences in ultrasonography metrics may correspond to differences in cognitive abilities, Further research is needed to examine these possibilities.

Gender effects are less frequent than hemispheric effects, with about 22% of comparisons being significantly different. In the instance of significant effects, females show higher scores in comparisons associated with the middle cerebral arteries. Males show higher values in comparisons that involve the internal carotid and vertebral arteries. A significant gender effect in the left hemisphere did not necessarily translate into a significant gender effect in the right hemisphere. The majority of gender effects for both hemispheres are observed in 10 year-olds in the left hemisphere, followed by the right hemisphere in the same age group. Although gender effects have been reported by some studies [10] but not others [15], the factors that drive these effects remain to be studied.

Machine learning classification models were used to examine whether we can distinguish children from adults from doppler ultrasonography indices. Imbalanced classification (i.e., in our data a large sample of children compared to adults) is commonly resolved by applying oversampling or undersampling techniques [43,45]. For comparison, we provide results of machine learning models with and without the application of these techniques (Table 3). Results show that oversampling improves classification models. The overall F1 score becomes higher with a lower standard deviation suggesting that it is more stable. This could be explained by the fact that such techniques may lower the variance of the classifier. Moreover, weak models which initially accurately classified the majority class (i.e., children), started to distinguish the minority class (i.e., adults) after sampling techniques were applied. By adjusting class distribution machine learning models become consistent and are able to detect dependencies in each class, providing further support for the application of oversampling or undersampling techniques [43,45].

Age group classification shows results, which are significantly different from random guesses. Although classification models are close in terms of standard deviation, model performance scores were significantly different from each other, with the model built on data from males performing with the highest accuracy. Our metrics show that scores from males have stronger relation with age in middle and internal carotid arteries than scores from females. They also show the strongest bivariate relations with age and lack of hemispheric asymmetries in the internal carotid arteries render it a more stable artery for building models. Indeed, this is confirmed with statistical analyses related to feature importance that shows internal carotid artery values are the strongest contributors in the classification models for both males and females (Fig 4). The features with the lowest importance are observed in the middle carotid artery, consistent with increased hemispheric variability we observe particularly in younger age groups. Metrics from the vertebral artery show high importance for models classifying female participants, consistent with fewer hemispheric differences observed for females in this blood vessel. The model that considers the total sample shows common important features with the model of males in the right internal carotid artery and with the model of females in the left vertebral artery. Past studies that considered age classification mainly examine digital images of faces [55,56]. Further, in these past studies neural networks were used to predict age groups based on face images or features extracted from them during pre-processing, thus this is the first study to consider age classification using ultrasonography metrics.

In interpreting the data from the current study, we point to three considerations. First, our sample is imbalanced with many more children compared to adults, therefore we have used recommended techniques for oversampling and undersampling for cross-validation. Second, we did not screen for medical conditions or other vital signs, because children were recruited from public schools and were attending regular classes. Although teachers, parents or school psychologists did not self-report a disability and neurodevelopmental disorder, we cannot eliminate the possibility that some children may have had a disability or medical condition that was undetected or unreported. Third, an inherent limitation of Doppler ultrasonography is reproducibility as images are reproduced after echoes between the distance from the probe to the objective, and observations are operator dependent. We have used a single operator for measurements; however, further research is needed to replicate and verify the applicability of the current models in different samples.

## Conclusions

Our data present for the first time individual differences related to age, gender, and hemisphere from three blood vessels in the same large sample of school-aged children and young adults. This is also the first machine learning study that demonstrates the feasibility of predictive and classification models. Findings demonstrate more similarities than differences as a function of gender and hemisphere, however, the existent significant differences are congruent with results observed in machine learning classification models, which show high accuracy. Our developmental scores and machine learning models can inform theoretical models of development and benefit future research and clinical practice in typical and atypically developing samples of children, such as those with neurodevelopmental disorders or vascular diseases. Applications may also be possible in educational and forensic sectors. Critically, the current study raises awareness of the possibilities machine learning in this field can offer and points to further directions for research that would replicate and clarify variability observed as a function of age, gender, and hemisphere.

## Supporting information

S1 Table Best regression models show mean absolute error scores. (XLSX)

S1 Appendix Materials and methods on quantitative age prediction using regression models. (DOCX)
