# Supplementary material for: Effects of age, gender, and hemisphere on cerebrovascular hemodynamics in children and young adults: Developmental scores and machine learning classifiers
Source: PLoS One. 2022 Feb 4;17(2):e0263106. doi: 10.1371/journal.pone.0263106 (PMC8815867; doi:10.1371/journal.pone.0263106)
Supplement: S1 Appendix — (DOCX) [file pone.0263106.s002.docx]

**SUPPLEMENTARY INFORMATION FOR:**

**Effects of age, gender, and hemisphere on cerebrovascular hemodynamics in children and young adults: Developmental scores and machine learning classifiers**

Authors: Marie Arsalidou^1,2^, Nikolay Skuratov^3^, Evgeny Khalezov^1^, Alexander Bernstein^3^, Evgeny Burnaev^3^, Maxim Sharaev^3^,

Affiliations:

1 - HSE University, Moscow, Russian Federation

2 - York University, Toronto, Canada

3 - Skolkovo Institute of Science and Technology, Moscow, Russian Federation

**Corresponding author:** Marie Arsalidou, HSE University, Moscow, 4 Armyansky Pereulok, Building 2, room 406, Moscow, 101000, Russian Federation. Email: [marie.arsalidou@gmail.com](mailto:marie.arsalidou@gmail.com); [marsalidou@hse.ru](mailto:marsalidou@hse.ru), [arsalido@yorku.ca](mailto:arsalido@yorku.ca)

**S1 Appendix**

**Materials and methods**

**Quantitative age prediction with regression models.**

Age in years is taken as the target variable. A standard pipeline of data processing was followed to achieve reproducibility of experimental results[1]. The pipeline consists of feature selection and dimensionality reduction, various machine learning models fitting and their hyperparameters search. To select the best model and assess its accuracy we used cross-validation.

Dimensionality reduction is a traditional method used to reduce correlations among features, which could result in overfitting and increased computational time. To apply dimensionality reduction we used both feature extraction and feature selection methods. To extract features we applied Principal Component Analysis (PCA)[2] and Locally Linear Embedding (LLE)[3]. For feature selection we used ready-to-use Python routines SelectKBest and SelectPercentile from the Scikit-learn library[4].

After feature selection and extraction, we considered various predictive models[5-7] and non-parametric estimation of confidence[8]. The most typical state-of-the-art approaches, which usually show good results in practice were applied: Linear Regression[9], Lasso Regression[10], Ridge Regression[11], K-Nearest Neighbours Regression[12], XGBoost Regression[13], Support Vector Regression[14], Random Forest Regression[15].

During model fit we searched for optimal model hyperparameters. This was done by grid search across different combinations of models, model parameters and dimensionality reduction procedures. We selected the best combination based on prediction error, and tested it on a validation dataset. To estimate the error the majority of previous studies used Root Mean Square Error (RMSE). However, RMSE is very sensitive to outliers[16] and to correct for that we measure accuracy using Mean Absolute Error (MAE):

$MAE=\frac{1}{n}\sum_{i=1}^{n} \left| x_{i}-x \right|$ (1)

After the best hyperparameters were selected, we fixed the structure of the resulting model and performed a final evaluation of its prediction accuracy by the “Shuffled K-fold” cross-validation approach[17]. Specifically, the dataset was randomly shuffled and then split into k groups. One of the groups was selected as a testing dataset, and all other groups were combined together and considered as a training dataset; the proposed model was fitted to the training data and test data were predicted with the obtained model; lastly, the predicted labels were compared to the original ones and the evaluation metric was calculated. This procedure was repeated for each group, after which a mean metric value was calculated for all k groups.

**Results**

**Predictive models: Regression results.**

Examples of significant dependencies particularly among Doppler ultrasound features are illustrated in Figure 1 of the manuscript: histograms are on the diagonal and scatter plots for corresponding pairs of features are given off the diagonal. Dependencies among features are observed, such as the dependency between vessel diameter and blood velocity of the same vessel in the same hemisphere. This indicates that some of these features could be redundant and these features can lead to instability of our model. To address this issue we applied methods of dimensionality reduction and feature selection.

Results show that hemodynamic indices can be used to estimate a child’s age within mean absolute error of 0.82 ± 0.06 for the whole sample (Table S1). The model can predict a child’s age with about 10 months accuracy by Lasso Regression and SelectKBest(f_classif) feature selection procedure. Mean absolute error of separate predictions is equal to 0.819 ±0.079 for males and to 0.799 ± 0.099 for females (Table S1). Best models are Random Forest Regression and SelectKBest(f_classif) for males; Support Vector Regression and SelectPercentile(f_classif) for females.

**S2 Table. Best regression models showing MAE scores.**

| Model | Task | Mean MAE | STD MAE |
| --- | --- | --- | --- |
| Lasso | All | **0.817** | **0.064** |
| Lasso + SelectKBest(f_classif) | All | 0.818 | 0.057 |
| Lasso + PCA | All | 0.820 | 0.066 |
| Ridge + PCA | All | 0.820 | 0.066 |
| LR + PCA | All | 0.821 | 0.066 |
| RFR + SelectKBest(f_classif) | Male | **0.819** | **0.079** |
| SVR+ SelectKBest(f_classif) | Male | 0.827 | 0.077 |
| RFR | Male | 0.831 | 0.076 |
| Ridge | Male | 0.836 | 0.094 |
| XGB + SelectKBest(f_classif) | Male | 0.837 | 0.085 |
| SVR + SelectPercentile(f_classif) | Female | **0.799** | **0.099** |
| KNR+ SelectPercentile(f_classif) | Female | 0.807 | 0.121 |
| RFR + SelectPercentile(f_classif) | Female | 0.807 | 0.101 |
| SVR+ SelectKBest(f_classif) | Female | 0.810 | 0.117 |
| RFR + SelectKBest(f_classif) | Female | 0.813 | 0.096 |

Regression methods: Lasso - Lasso regression, Ridge - Ridge Regression, LR - Linear Regression, KNR - K-Nearest Neighbours Regression, XGB - XGBoost Regression, SVR - Support Vector Regression, RFR - Random Forest Regression. Dimensionality reduction methods: None - original data without dimensionality reduction, SelectKBest, SelectPercentile, PCA - Principal Component Analysis, LLE - Locally Linear Embedding. Tasks: All - Experiments with total sample. Best models highlighted in bold.

**Discussion**

Ultrasonography metrics are used for the first time with machine learning to predict age and classify age groups. Predictive models suggest that a child’s age is reliably predicted within 10 months using ultrasonography indices. Comparing with previous neuroimaging studies our mean absolute prediction error is lower than studies that used electroencephalography (1.22 years = 14.63 months)[18] and magnetic resonance imaging (1.1 years = 13.2 months)[19]. Specifically, past studies used smaller sample sizes (5 to 19 years N=394)^19^ or discontinuous ages (i.e., 5, 7, 12, 14, 16, and 18, N = 702)[18]. A higher predictive precision in our study may be due to a narrower continuous age range and a larger sample size. Separating a dataset according to gender gave us a slight increase in accuracy of age prediction for males, in all other terms prediction strength is comparable for all three datasets. Although our sample consists of a large sample of continuous age groups, the disproportionate sample sizes pose further consideration. Specifically, we compared our results to a simple logistic regression model to find that a similar error of 10 months is achieved, suggesting that the machine learning model may not make a substantial contribution to prediction. According to a recent meta-analyses predictive machine learning models are often not superior from logistic regression for clinical predictions[20]. An improved approach for addressing this issue is to combine quantitative prediction models, with classification models to verify convergence in model parameters.

References

1. Sharaev M, Andreev A, Artemov A, Burnaev E, Kondratyeva E, Sushchinskaya S *et al.* Pattern recognition pipeline for neuroimaging data. In: *IAPR Workshop on Artificial Neural Networks in Pattern Recognition*. 2018, pp 306–319.
2. Wold S, Esbensen K, Geladi P. Principal component analysis. *Chemom Intell Lab Syst* 1987; **2**: 37–52.
3. Roweis ST, Saul LK. Nonlinear dimensionality reduction by locally linear embedding. *Science (80- )* 2000; **290**: 2323–2326.
4. Pedregosa F, Varoquaux G, Gramfort A, Michel V, Thirion B, Grisel O *et al.* Scikit-learn: Machine learning in Python. *J Mach Learn Res* 2011; **12**: 2825–2830.
5. Kuleshov A, Bernstein A, Burnaev E. Manifold learning regression with non-stationary kernels. In: *IAPR Workshop on Artificial Neural Networks in Pattern Recognition*. 2018, pp 152–164.
6. Kuleshov A, Bernstein A, Burnaev E. Kernel regression on manifold valued data. In: *2018 IEEE 5th International Conference on Data Science and Advanced Analytics (DSAA)*. 2018, pp 120–129.
7. Belyaev M, Burnaev E, Kapushev E, Panov M, Prikhodko P, Vetrov D *et al.* Gtapprox: Surrogate modeling for industrial design. *Adv Eng Softw* 2016; **102**: 29–39.
8. Burnaev E, Nazarov I. Conformalized kernel ridge regression. In: *2016 15th IEEE international conference on machine learning and applications (ICMLA)*. 2016, pp 45–52.
9. Neter J, Kutner MH, Nachtsheim CJ, Wasserman W. Applied linear statistical models. 1996.
10. Tibshirani R. Regression shrinkage and selection via the lasso. *J R Stat Soc Ser B* 1996; **58**: 267–288.
11. Hoerl AE, Kennard RW. Ridge regression: Biased estimation for nonorthogonal problems. *Technometrics* 1970; **12**: 55–67.
12. Altman NS. An introduction to kernel and nearest-neighbor nonparametric regression. *Am Stat* 1992; **46**: 175–185.
13. Chen T, Guestrin C. Xgboost: A scalable tree boosting system. In: *Proceedings of the 22nd acm sigkdd international conference on knowledge discovery and data mining*. 2016, pp 785–794.
14. Drucker H, Burges CJ, Kaufman L, Smola A, Vapnik V. Support vector regression machines. *Adv Neural Inf Process Syst* 1996; **9**: 155–161.
15. Liaw A, Wiener M, others. Classification and regression by randomForest. *R news* 2002; **2**: 18–22.
16. Fedintsev A, Kashtanova D, Tkacheva O, Strazhesko I, Kudryavtseva A, Baranova A *et al.* Markers of arterial health could serve as accurate non-invasive predictors of human biological and chronological age. *Aging (Albany NY)* 2017; **9**: 1280.
17. Kohavi R, others. A study of cross-validation and bootstrap for accuracy estimation and model selection. In: *Ijcai*. 1995, pp 1137–1145.
18. Vandenbosch MMLJZ, van ’t Ent D, Boomsma DI, Anokhin AP, Smit DJA. EEG‐based age‐prediction models as stable and heritable indicators of brain maturational level in children and adolescents. *Hum Brain Mapp* 2019; **40**: 1919–1926.
19. Franke K, Luders E, May A, Wilke M, Gaser C. Brain maturation: predicting individual BrainAGE in children and adolescents using structural MRI. *Neuroimage* 2012; **63**: 1305–1312.
20. Christodoulou E, Ma J, Collins GS, Steyerberg EW, Verbakel JY, Van Calster B. A systematic review shows no performance benefit of machine learning over logistic regression for clinical prediction models. J. Clin. Epidemiol. 2019; **110**: 12–22.
